# A Review on Biotechnological Approaches Applied for Marine Hydrocarbon Spills Remediation

**DOI:** 10.3390/microorganisms10071289

**Published:** 2022-06-25

**Authors:** Farzad Rahmati, Behnam Asgari Lajayer, Najmeh Shadfar, Peter M. van Bodegom, Eric D. van Hullebusch

**Affiliations:** 1 Department of Microbiology, Faculty of Science, Qom Branch, Islamic Azad University (IAU), Qom 37185364, Iran; farzad.rahmati_script@yahoo.com; 2 Department of Soil Science, Faculty of Agriculture, University of Tabriz, Tabriz 5166616422, Iran; 3 Department of Biotechnology, Faculty of Biological Sciences, Alzahra University, Tehran 1993893973, Iran; n.shadfar@student.alzahra.ac.ir; 4 Department of Environmental Biology, Institute of Environmental Sciences, Leiden University, P.O. Box 9518, 2300 RA Leiden, The Netherlands; p.m.van.bodegom@cml.leidenuniv.nl; 5 Institut de Physique du Globe de Paris, Université Paris Cité, CNRS, 75005 Paris, France

**Keywords:** oil spills, bioremediation, biostimulation, bioaugmentation, biosurfactants, petroleum hydrocarbons

## Abstract

The increasing demand for petroleum products generates needs for innovative and reliable methods for cleaning up crude oil spills. Annually, several oil spills occur around the world, which brings numerous ecological and environmental disasters on the surface of deep seawaters like oceans. Biological and physico-chemical remediation technologies can be efficient in terms of spill cleanup and microorganisms—mainly bacteria—are the main ones responsible for petroleum hydrocarbons (PHCs) degradation such as crude oil. Currently, biodegradation is considered as one of the most sustainable and efficient techniques for the removal of PHCs. However, environmental factors associated with the functioning and performance of microorganisms involved in hydrocarbon-degradation have remained relatively unclear. This has limited our understanding on how to select and inoculate microorganisms within technologies of cleaning and to optimize physico-chemical remediation and degradation methods. This review article presents the latest discoveries in bioremediation techniques such as biostimulation, bioaugmentation, and biosurfactants as well as immobilization strategies for increasing the efficiency. Besides, environmental affecting factors and microbial strains engaged in bioremediation and biodegradation of PHCs in marines are discussed.

## 1. Introduction

Petroleum hydrocarbons (HCs) or crude oil are principal pollutants of aquatic ecosystems due to transportation accidents, excavation, natural oil penetration, ruptured pipelines, and regular cleaning ways of storage tankers [1]. In December 1907, the first enormous marine oil spill happened near the Isles of Scilly (The southwestern tip of Cornwall, England) and a huge amount of paraffin (approximately 7400 tons) polluted the coast. Since that first oil spill, about 7.5 million tons of crude oil have been released into marine environments [2]. In just one case alone, over seven hundred thousand tons of oil were released near the coast of Mexico through a disaster called Deepwater Horizon [3]. Oil spills can have broad economic and environmental impacts. In the 1989 oil spill, more than 250,000 seabirds were slaughtered during the Deepwater Horizon disaster, which generated a financial loss of $61 billion [4].

Following an oil spill, the biological and physicochemical features of crude oil undergo changes (e.g., through weathering and degradation). For instance, viscosity density and nickel “vanadium” sulfur concentration, and asphaltenes are subject to change during the initial months. Besides, crude oils are recognized to form sticky water-in-oil emulsions, whereas asphalt-free products do not produce emulsions. Microorganisms like algae, fungi, archaea, and bacteria can degrade crude oil in marine environments. Table 1 depicts the high metabolic versatility of microorganisms that mostly target substrates like n-alkanes and polycyclic aromatic hydrocarbons (PAHs). Microorganisms play a pivotal role in reducing the accumulation of metabolites resulting from PAHs degradation. While many of these microorganisms are anaerobic, other groups rely on oxygen for their metabolism, showing that there is no special pattern in terms of metabolic pathways and genomes. Crude oil-degrading microorganism genomes and physiologies have been intensively studied during the last decade. The investigations revealed that these bacteria and fungi are biosurfactant producers, sulfate-reducing, resistant to potentially toxic metals, extremophiles, and hyperthermophiles [5]. They constitute a mix of generalist and specialist microorganisms, based on their abilities to colonize a wider set of carbon sources and a narrow range of HCs [6]. Microorganisms have employed certain strategies to improve the degradation pathways and to increase bioavailability (Such as surfactant production, adhesion to the phenanthrene crystals, and chemotactic responses; discussed in more detail in next sections) of weathered HCs in order to use them as energy and carbon sources [7].

Bioremediation and biodegradation technologies have been applied to remove deep-sea oil spills since a half-century ago. Bioremediation engages in treating crude oil with hydrocarbon-degrading microbes with a vast number of enzymes. This method is a low-technology technique, viable, with relatively low cost, which breaks down the pollutants in a short period (Between 1 and 4 weeks) and its application is prevalent [36]. Consequently, these technologies have been the practical methods for restoring aquatic ecosystems. Nonetheless, the most critical problems of these methods are the presence of unpredictable and uncontrollable parameters, causing a significant reduction in remediation efficiency, and the loss of effective microbial strains because the ocean is a highly mobile and open system. These unpredictable factors include environmental factors like the presence of oxygen and nutrients, temperature, pH, and sediment type. Besides, the availability of contaminants to the microbial population and the existence of a microbial population capable of degrading the pollutants are other uncontrollable parameters that may reduce the bioremediation process efficiency. Immobilized microbial technology has been a popular technique to create enhanced tolerance to the environment, limiting microbial loss, high cell concentration, simple operation, reuse, and high biodegradation efficiency of HCs [37,38]. Polyvinyl alcohol boric acid embedding and calcium alginate embedding (Sodium alginate–calcium chloride) are the most prevalently used methods of embedding immobilization [12]. A comparison between embedded immobilization by polyvinyl alcohol-boric acid and calcium alginate embedding method showed that the first one has a longer service life and higher mechanical power [39]. Polyvinyl alcohol boric acid embedding after immobilization decreases the activity of microorganisms owing to challenges in ball forming derived from polyvinyl alcohol and saturated boric acid toxicities [27]. Calcium alginate embedding is a pioneering technique to provide better functioning of encapsulated cells, that is not toxic to the microorganisms of the embedded materials, and has mild reaction conditions [40,41]. Many articles have reviewed oil spills worldwide and have elaborated on the biotechnological and chemical strategies used to remove crude oils from marine surfaces [1,6,36]. However, the majority of these review articles focused on the materials and agents applied, e.g., discussing the various types of particles, biomass sorbents, surfactants, separators, gelators, and aerogels considered in different bio-based methods. Nonetheless, previous reviews have not aimed at discussing the pivotal role of microorganisms and their adaptations. It stresses more consideration to unfold the microbial aspects of this issue.

This review focuses on the microbial aspects of the bioremediation techniques rather than on chemical-based technologies in oil spill remediation. This review specifically focuses on the most recent biotechnological strategies such as biostimulation, bioaugmentation, and use of biosurfactants with their advantages and challenges. Additionally, available techniques involved in cell immobilization for increasing the efficiency of bioremediation will be discussed. Finally, the identified microorganisms involved in bioremediation and biodegradation, and specifically those engaged in bioaugmentation, as well as potential environmental factors affecting the bioremediation by microorganisms, will be discussed.

## 2. Microorganisms Involved in the Removal of Oil Spills from Marine Surfaces

Yeasts, fungi, and bacteria have the potential to use the various HCs in crude oil and derived products. For instance, following the Deepwater Horizon disaster (20 April 2010), the bacterial community identified was prone to succession [42,43]. At first, a primary enrichment of *Pseudomonads* and *Oceanospirillales* prevailed, followed by a change in dominance to *Cycloclasticus*, *Methylotrophs*, *Colwellia*, and *Pseudoalteromonas.* Genes associated with the transportation of iron or phosphorus and nitrogen-based compounds and associated metabolisms such as nitrate reduction were transcribed [44]. Correspondingly, total petroleum hydrocarbon and ammonium concentrations on the surfaces of the sediment were correlated with denitrification-related genes that were highly transcribed [45]. The gene expression for sulfite reduction and sulfur cycling was raised when O_2_ levels stayed high, however, the relevant mechanism is still unclear [46]. Several months after the DWH (Deepwater Horizon) spill, the sediment of the wellhead area region was enriched with *Chloroflexi*, *Firmicutes*, *Methylotrophic*, and *Actinobacteria* [47]. An exploratory survey was conducted and showed that—after a decade—the microorganisms of the sediments had returned to pre-spill composition [10].

While aliphatic HCs can be properly biodegraded in nature, this is completely different for branched-chain and long-chain HCs [48,49,50]. Moreover, with increasing pH and increasing salinity, the rate of degradation decreases, and that is why aliphatic HCs are hardly degraded in crude oil under hypersaline circumstances [8]. Salinity not only influences the diversity and growth of microorganisms, but also has a direct effect on biodegradation, and enzymes engaged in HCs degradation undergo some changes and lose their functioning. This inhibition is reversible when the microorganisms go back to normal conditions (moderate salinity level). Knowledge about molecular mechanisms and pathways of HCs degradation at high salinity is rare and only recently there have been a few reports explaining genes, enzymes, and breakdown steps for some HCs. These investigations have clearly shown that degradation of oxygenated and non-HCs by halophilic and halotolerant bacteria occurs by pathways similar to those found in non-halophiles [51]. In seasons with high temperatures and evaporation, salinity tends to increase and HCs degradation rate is remarkably reduced in deep-sea water [52]. During recent years many investigations have been performed on PAHs bioremediation and resources (Table 2). Several bacterial enzymes with various catabolic productivity allied with PAHs degradation have been identified and various biochemical pathways for PAH degradation (Such as naphthalene, phenanthrene, anthracene, and acenaphthene) have been introduced. Furthermore, some PAH catabolic operons have been sequenced, and their regulatory mechanism for PAH degradation has been known. In the environment, microorganisms are able to degrade different types of PAHs including quinoline, benzothiophene, and benzofuran through biochemical pathways. Nonetheless, a more detailed study is required to determine what is going on in a PAH-contaminated environment. Besides, many aspects of PAHs bioremediation have remained unknown or insufficient, which demands prospective attention. There is little knowledge on the transmembrane trafficking of PAHs, genes, enzymes, and molecular mechanisms of PAHs degradation in anaerobic conditions or high salinity and acidic environments. During the last decade, many studies have focused on *Fusarium* species ability in metabolizing the hydrocarbon chrysene to help to identify fungal strains displaying crude oil degradation potential under saline conditions [53,54].

## 3. Bioremediation and Affecting Factors

The first modern bioremediation use refers to six decades ago when George Robinson recruited the microbes to consume oil spills on the coast of Santa Barbara, California [64]. However, this technology was not important for the removal of HCs and other hazardous compounds like potentially toxic metals until the early 1980s [64]. Generally, bioremediation is a process associated with microorganisms and their metabolites to remove the different contaminants from aquatic ecosystems and sediment. Particularly, indigenous microorganisms in sediment are the main players in bioremediation because they can transform organic compounds into simple inorganic compounds or into their constituent elements through a process called mineralization [65]. Microorganisms in the bioremediation process have the potential to eliminate, decrease and transform oil contaminants into natural compounds found in air, sediments, water, sludge, and soil. Often, bioremediation co-occurs with detoxification, when microorganisms remove or immobilize waste materials by altering mineralization, and transformation [66]. For microbial cell reproduction and viability, the presence of energy is necessary, which is obtained in the bioremediation process through redox reactions like respiration. In this energy supplying system, the presence of nutrients and energy sources, including electron donor and acceptor (Such as carbon dioxide, oxygen, iron (III), and sulfate) is essential [67]. In HCs bioremediation, the availability of a microbial strain or consortia with suitable metabolic capabilities is the most challenging. Microbial communities adapt, change genetically and selectively enrich within hours of exposure to HCs. As a result, higher biodegradation rates are achieved than in communities with no introduction of HCs contamination [68]. In bioremediation of HCs polluted sites, isolation of appropriate numbers of special HCs degraders from an environment is a justification for effective bioremediation. On the other hand, usually, consortia of different strains are required in the bioremediation of oil spills because it is made of a mixture of compounds, and one strain can metabolize only a limited range of HC substrates. Bioremediation relies on nutrient presence and optimal site properties that support biological functions. For example, microbial growth is affected when exposed to a high concentration of the contaminants owing to the presence of high toxins. In contrast, low concentration can block the induction of bacterial enzymes [64]. Additionally, an optimum availability of water (between 14% and 27% moisture) and pH (in the range of 5.5–7.8) in the environment is vital for the proliferation and growth of microbial cells [69]. The biodegradation efficiency, to an extent, rises with increasing temperature and decreases with reducing temperature [69]. Nutrient availability (e.g., through organic substrate amendments) is also needed for cell growth, division, and electron donor as a biostimulant [64]. Finally, contaminant bioavailability is another factor impacting the bioremediation process. Bioavailability for microbial reactions is lower for contaminants that are more sorbed to solids attached in matrices of molecules in contaminated sites, more widely diffused in macropores of soil and sediments, or are present in non-aqueous phase liquid (NAPL) form [64]. In the next sections, we discuss the role of environmental factors affecting the bioremediation process.

## 4. Biologically Based Solutions for Ashore and Marine Pollution

**Figure 1:**
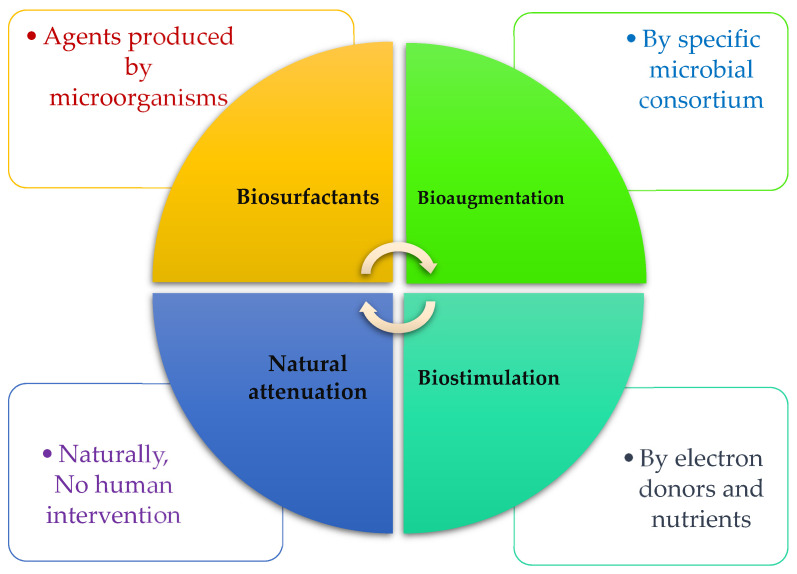
The most prevailing bioremediation techniques are used for HCs degradation.

In the following sub-section, we review three common bioremediation technologies that are practical for eliminating oil spills in marine environments by microorganisms. In addition, factors and strategies for increasing the efficiency of technologies will be discussed. Figure 1 depicts what agents are responsible for three techniques of biosurfactant, biostimulation, and bioaugmentation.

### 4.1. Bioaugmentation

Generally, adding oil-degrading bacteria to the existing microbial community is called bioaugmentation [70]. Bioremediation activities intend to boost the degradation efficiency that is performed by adding exogenic microorganisms. Bioaugmentation is considered a “polishing-up” or “accomplishing” process to deal with the very slow initial degradation of a fresh oil spill due to the very high concentrations of the originally spilled oil and its concomitant high abundance of toxic compounds. Usually, non-native microorganisms are looking for a suitable amount of biosurfactant when they are exposed to perilous oil spills to avoid detrimental impacts due to the toxicity of the spill. The HCs non-indigenous and indigenous degrading bacteria employ intracellular enzymes that permit bacteria to transform the HCs into another food source. Usually, on a HCs-containing culture medium or at the site of a spill in bioreactors, oil-degrading microbes are concentrated, microbial agents. These agents aim to prepare the influenced region with a significant oil-degrading microbial inoculum [71]. Therefore, bioaugmentation helps increasing the number of HCs degraders to a point that the spilled oil is used as the principal energy source. The most prevalent used options for bioaugmentation are the introduction of genetically engineered bacteria and the addition of biodegradation relevant genes packaged in a vector to be transferred into indigenous microorganisms by conjugation, as well as the addition of a pre-adapted consortium and a pre-adapted pure bacterial strain [72]. Screening followed by tailoring a competent microbial community for a particular site is a prerequisite for bioaugmentation. The first screening or selection step should pay attention to the metabolic capacities of the microorganism and vital characteristics that keep the cells active and alive under undesired environmental conditions. Prior knowledge about the microbial communities inhabiting the target site is an excellent strategy to select effective communities. Sometimes in co-contaminated sites, where both organic and metal contaminations are found, the best strategy is to take advantage of multi-component systems, i.e., a creating led microbial consortium is more beneficial than single-component systems [73]. Additionally, using a microbial consortium is more practical compared with a pure culture because it provides the metabolic diversity and reliability needed for field applications. Table 3 indicates relevant microbial consortia in the bioaugmentation. Some researchers using selected native strains reported full degradation of diesel oil and phenanthrene; an overall decrease of about 75% of the total HCs, and a decrease of 60% of isoprenoids in 40 days [74,75]. In 2009, Li et al. [76] confirmed that using indigenous microbial consortia (three bacteria including *Bacillus* sp., *Zoogloea* sp., and *Flavobacterium* with five fungal strains including *Aspergillus niger*, *Phanerochaete*, *Cuuninghamella* sp, *Chrysosporium, Alternaria alternate*, and *Keissler, Penicillium chrysogenum*) enhanced the degradation of PAHs in water by 41.5%. The biomass to be employed as inoculum for bioaugmentation is produced in bioreactors, and transferring of cultures to the site is often challenging. After being exposed to environmental stresses, in contrast to the optimum conditions of culturing, the introduced population starts reducing quickly. Fluctuations and shifts in some environmental factors like temperature, pH, moisture, as well as toxic pollutant levels, and depletion of nutrients influence microbial growth. Goldstein et al. argued that in natural systems, unlike their potential in cultures, inoculant usually cannot degrade organic pollutants [77]. Problems regarding the adaptation of the inoculated microorganisms, the rivalry between introduced and indigenous biomass, insufficiency of the substrate, and the use of other organic substrates are the most important reasons for the failure of degradation in natural systems [71].

### 4.2. Biostimulation

In many situations, environmental criteria can be altered to boost the process of biodegradation. In marine crude oil spills, it is challenging to extend the life span of populations of HCs-eating microbes, owing to the problematic use of nutrients, and several native species can be killed or weakened because of oil toxicity in the spill region [88]. This toxicity is an inhibitory factor for stimulating the remaining indigenous bacteria and fungi. In this situation, biostimulation—helping optimize the environmental factors—is an appropriate strategy for degrading the HCs when the indigenous microbes are retained, and toxicity concentration is decreased [88]. Biostimulation may be more beneficial than bioaugmentation, as it stimulates indigenous bacteria that were already more competitive. It has been reported that if microorganisms growth is stimulated in the spill area, the HCs degrading efficiency may reach 70% during 30 days of incubation [89].

Nutrients are vital components of an effective biostimulation of HCs, as some nutrients may become an inhibiting factor, negatively affecting the biodegradation process [88]. The most prevalent additives that promote bacterial growth in a community are phosphate salts and nitrate [90]. The main aim is to boost the metabolism of autochthonous HCs degraders through in situ addition of limiting factors, which results in a quicker rate of the HCs degradation. The application of biostimulation in HCs bioremediation has been widely reported [34]; however, this approach also has its limitations and challenges [91]. For example, in soils, the use of inorganic fertilizer is challenged by the large cost of bioremediation and the likely chance of eutrophication [92].

### 4.3. Biosurfactants

Biological active surface molecules produced by microorganisms with a huge range of applications owing to their specific characteristics, minute toxicity, and biological admissibility are called biosurfactants [93]. These products as additives aim to produce petrochemicals and organic chemicals that are more accessible. Biosurfactants are amphiphilic compounds and able to alter the cell surface of microorganisms and increase the hydrophobic substance surface area. Microorganisms thanks to their ability of using hydrocarbon waste as raw materials can produce biosurfactants. One of the unique properties of biosurfactants is tolerance to environmental extreme conditions like ionic strength, acidity, temperature, salt concentration, demulsifying-emulsifying ability, and anti-inflammatory potential due to surface and interface activity [94]. Recent investigations have reported that lichenysin biosurfactant produced by *Bacillus licheniformis* was hardly impacted by Ca, NaCl concentrations, or temperature (up to 50 °C) and pH (4.5–9.0) [95]. Additionally, lipopeptides produced by *Bacillus subtilis* are stable at −15 °C, and beyond autoclavable temperature (121 °C) and concentrations, greater than 15% of NaCl when stored for six months [95]. An ideal biosurfactant decreases the surface tension of water. For instance, biosurfactants like surfactin, rhamnolipid, and sophorolipids produced, respectively, by *B. Subtilis*, *P. aeruginosa*, and *Candida bombicola* lower the surface tension [96]. Biosurfactants are productive and efficient, and their critical micelle concentration (CMC) is ten to forty times lower than that of chemical surfactants. Hence, less biosurfactant is required to decrease the surface tension [97]. Biosurfactants also have antioxidant, antimicrobial, and anti-inflammatory activities. For instance, recent research suggested that the polyanionic surfactant named emulsan produced by *Acinetobacter calcoaceticus* has indicated lethal concentration 50 (LC50) against other microbes like *Photobacterium phosphoreum*, which is much less than *Pseudomonas rhamnolipids* [98]. Biodegradability and environmental toxic friendly nature are two other properties of biosurfactants, as most biosurfactants are easily degradable.

Various microorganisms produce biosurfactants involved in the bioremediation of HCs. Table 4 shows economic and prevalent biosurfactants produced by different microorganisms. Rhamnolipid in two forms of mono and di-rhamnolipid is a type of biosurfactant produced by *P. aeruginosa* [99]. A correlation exists between the type of surfactant and the type of HCs that are degraded. For example, rhamnolipids produced by *P. aeruginosa* specifically degrade hexadecane. Many studies were done on phenanthrene degradation by chemical surfactants, and findings have proven that increased phenanthrene degradation is along with bacterial isolates producing a non-ionic surfactant [100]. Based on a study, HCs degradation capacity was multiplied when it was mixed with a biosurfactant, like a combination of chemical surfactant ‘FinasolOSR-5′ with biosurfactant trehalose-5, 5′-dicorynomycolates [100]. It has been reported that PAHs are significantly degraded by bacteria producing sophorose lipids and glycolipids in less than a month because surface-active glycolipids increase the biodegradation of 2,4-dichlorophenolindophenol (DCPIP) when supplemented with HCs sites [101].

### 4.4. Cell Immobilization Techniques for Increasing Bioremediation Efficiency

Several techniques are available for immobilization of the microbial cells to increase the bioremediation efficiency by microorganisms. A carrier allows supplying moisture and aeration, better access to nutrients, and physical support for biomass, which increases the survival of the microbes [110]. To increase the viability and efficiency of the microbial cells, entrapment and encapsulation are two main techniques that accelerate the biodegradation process compared to uncoated cells.

Entrapment is an irremeable immobilization strategy for capturing cells inside fibers, and has been extensively studied. The systems provide enough protection by creating barriers around the immobilized microbial cells, therefore ensuring sustainable viability of the cell when the microorganisms are surrounded by the polymer [111]. Synthetic polymers like photo-cross-linkable resins and polyester as well as natural polymers like gelatin, collagen, and alginates are the most significant accessible matrices for entrapment. This process is designed with microbial cells within a solid network to allow the penetration of the substrate when inhibiting the cells from scattering into the medium. Owing to moderate required conditions and easy application of alginate gel polymer, many studies have reported practical use of this polymer for entrapment [111]. High cost, leakage of the cells, low loading capacity for the conjugation of biocatalysts into the matrix, limitations in disseminations, and attrition of biocarriers caused by high mechanical power are the most significant demerits of this method [112]. Nonetheless, this method is the most prevalent technique.

Encapsulation can reduce the concentration of toxic substances, control the availability of the nutrients, decrease the cell membrane damage, and protects the microbial cells from predation and competition [112]. Gellan gum, polyurethane, alginate, polyvinyl alcohol gel, kappa-carrageenan, gelatin, agar, agarose, and acrylate copolymers have been the studied components for immobilization and encapsulation of the HCs-degrader bacteria [71]. A study conducted by Muslemy et al. [113] worked on one bacterial consortium encapsulation with gellan gum microbeads and showed that encapsulated cells revealed a shorter lag phase. Consequently, a higher HCs degradation rate was observed in comparison with normal form (Free cell) at equal microbial concentrations. Liu et al. (2009) investigated the ability of *Sphingomonas* sp. FG03 and *Acinetobacter* sp. XA05 strains for biodegradation of phenol in both free and encapsulated settings in marine ecosystems [114]. Findings showed that a mixture of two bacterial strains had better efficiency in phenol biodegradation than pure cultures, and had enhanced functions for the high concentration of phenols [114]. In a study, Yaohui and Mang [115] worked on the differences between biostimulation and bioaugmentation treatments in the bioremediation of HCs and encapsulated the microbes with peanut hull powder as a carrier and bulking agent. They reported that mass transfer rate of nutrients, oxygen, water, HCs, and nutrition for the microflora was accelerated [115]. Wang et al. argued that immobilization of the *Mucor* sp. F2 (MF) fungal consortium, *Mycobacterium* sp. B2 (MB) fungal consortium, and MB+MF with pyrene, respectively, increased by 159.1%, 59.9%, and 60.0% after incubation [116]. Dehydrogenase activity has also a considerable improvement when biocarriers are used. Keryn et al. [117] investigated the effectiveness of economically sustainable biocarriers (mussel shells, coir peat complex) for marine HCs remediation and reported an accelerated degradation in the immobilized forms compared with free cells.

Researchers have investigated different ways of combining biostimulation and bioaugmentation technologies for bioremediation. Researchers have assessed several combinations of consortium or single bacteria and fungi with biostimulators such as fertilizers, corn-steep-liquor, solid-waste-dates, and other materials containing N, K, and P and obtained satisfying results. The degradation efficiency reached 97% and 91% for 0.5% *w*/*v* crude oil in 4 weeks, respectively by solid-waste-dates and corn-steep-liquor as biostimulants and using single strain *Pseudomonas* [118]. The degradation efficiency is affected by the type of total petroleum HCs pollutant to be degraded as light crude oil is more degradable than heavy crude oil, and this process is faster. In a study, Arabian light crude oil (1000 ppm) was degraded within three weeks by a single strain of *Alcanivorax borkumensis* SK2, and the presence of biostimulators like 0.1 g/L NaNO_3_, 0.077 g/L KH_2_PO_4_, and 0.2 g/L NH_4_Cl [119]. Additionally, 94.4% efficiency was obtained for 10% *v/v* crude oil (Escravos light) in 8 weeks by *Aspergillus niger* and *Pseudomonas aeruginosa,* and the presence of K, N, P [119]. Another study treated water polluted with crude oil by a combination of biostimulation and bioaugmentation technologies by *A. niger* and *P. aeruginosa*. The researchers created four different conditions including (nutrient-free), A (nutrient N, P, K), B (nutrient-plus aeration), and C (nutrient-free, aeration, and agitation), and achieved efficiencies of 92.3%, 93.6%, and 94.4%, respectively, after 56 days, for total petroleum HCs degradation [120].

Cross-linking is another technique employing hydrogen bonds (Covalent) between microbial cells and inorganic support by agents. Chemical modification of the surface of support materials is the main element of this technique. Compared to the previous technique, cross-linking is less practical because covalent binding often cuts functional integrities in the microbial cell. However, this method has very high efficiency for the cell when the functional integrities remain without interruption. Many successes regarding this technique refer to the immobilization of yeast cells. For example, in two studies in this area, researchers reported a successful application of cross-linking in the immobilization of *Saccharomyces carlsbergensis* on porous silica beads [121] and *Saccharomyces amurcea* and *Saccharomyces cerevisiae* on zirconia ceramics [122].

## 5. Factors Engaged in Microbial Degradation

**Figure 2:**
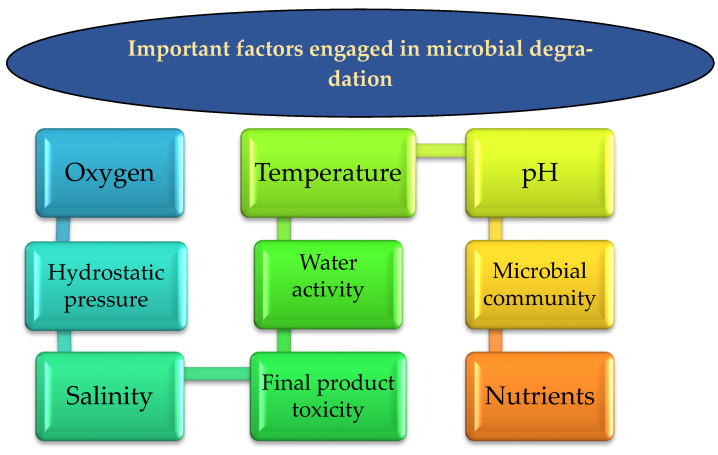
The most important factors impacting biodegradation by microorganisms.

The microbial efficiency in the biodegradation process of HCs depends on multiple environmental factors (Figure 2). In the next subsections, potential factors will be discussed in detail to what extent they can impact the microorganism functionalities.

### 5.1. Oxygen Bioavailability

Oxygen availability is an important factor that determine the pathways for hydrocarbon biodegradation. Under aerobic conditions, enzymes of the microorganisms can stimulate hydrocarbon removal by integrating oxygen atoms and producing alcohols. Furthermore, in this pathway, alcohol can be oxidized to produce carboxylic acids. Generally, alkanes represent a significant fraction of crude oils. The combination of alkanes with fumarate and secondary carbon triggers hydrocarbon hydrolysis at aerobic conditions. Alkane hydroxylases are a prominent investigated aerobic enzyme encoded by the almA and alkB genes, as well as p450 cytochrome [123,124]. In anaerobic conditions, however, the addition of fumarate to alkanes, leads to the synthesis of alkyl succinates, which is an important enzyme in anaerobic biodegradation [16]. The full metabolic pathways engaged in hydrocarbon catabolism are not yet clear under in situ conditions. Particularly, the impacts of environmental factors on activities and the pathways are not fully known. However, the availability of meta-omics data allows our imagination to visualize the process [125]. Hydrocarbon cleanup practices can make use of oxygen as a driver, e.g., by creating electron flows utilized by the “Oil-spill snorkel”. This innovative bioelectrochemical approach aims to stimulate the oxidative biodegradation of HCs in sludge and sediments of coastal regions [6]. The snorkel is inserted in the sediments and acts as a single conductive material. Making a bioelectrochemical connection between the polluted sediments and the overlying O_2_-containing water is the main task of the snorkel. Electrons derived from the oxidation of contaminants are taken by an electrode inserted in the sludge or sediments. By the snorkel, these electrons are transferred to the cathode (aerobic environment) where they reduce oxygen to form water [126].

### 5.2. Hydrostatic Pressure

Hydrostatic pressure (HP) is a natural attribute of the marine environment. HP increases linearly with depth and affects cell functions and structures, creating hypoxic conditions and decreasing enzymatic functioning, as it inhibits the membrane`s transportations (Le Châtelier’s principle). *Alcanivorax* spp. was the first and ubiquitous microorganism detected after the Deep Water Horizon oil spill disaster. For many years the impact of HP on the functioning of this microbe was studied [127,128], even though DWH deep-sea water and sediment samples indicated a high abundance of *Oceanospirillales* and a low abundance of *Alcanivorax* [129,130]. Nonetheless, a significant abundance of *Alcanivorax* after cultivation under atmospheric pressure was observed in oil-contaminated shores, and oil patches on the marine surface [131,132]. *Alcanivorax* strains have also been collected from marine enriched cultures obtained from 5000 m below the water level and HC-free. At such deep conditions, however, i.e., from an HP of 10 MPa onwards, the cultivation of three *Alcanivorix* species (*A. jadensis, A. dieselolei,* and *A. borkumensis*) stopped [6,133]. At those HP conditions, the ectoine production is energy-consuming and does not bring profitability to *Alcanivorax* growth, and the metabolic chain shifts from cytochrome oxidases to reductases [134]. These endothermic conditions also suggest that syntrophic interactions might be important for oil spill degradation at deep sea conditions.

The latest research reveals that HP also impacts the metabolic pathways of other oil-degrading microorganisms [6]. HP can influence macromolecule structures and chemical reactions, particularly of protein complexes with several subunits and weak non-covalent bonds. This is the case for protein matrices, ribosomes, and nucleic acids assembly [135,136], and changes are experienced at sub-lethal pressures. As a consequence, many functions like protein synthesis become inactive beyond a HP of 50 MPa, and the ribosome number falls dramatically [137]. Internal cell structures and cell membranes are damaged, and protein denaturation is interrupted when the microorganisms are exposed to 50 MPa HP [138].

Microbial cell membranes containing a high amount of mono-unsaturated fatty acids that are vital for the maintenance of sustainable and stable cell membrane homeostasis and fluidity [137]. The HP and other factors like low temperature in deep-sea waters increase the amount of mono-unsaturated fatty acids. Subsequently, when the accumulation of these mono-unsaturated fatty acids is high in the cell membranes, the cell membrane’s viscous properties and curvature elastic stress are positively affected. Still, it is not clear in what ways HP and other environmental factors increase the rate of cell membrane fatty acids. HP also influences transmembrane enzymes, and membrane transport systems, like cytochromes and ATPases, owing to direct impacts on the lipid environment on the membrane enzyme or its folding [139]. Potentially, compatibility with high HP stables energy production and proteins in microorganisms, probably to neutralize HP-related stress impacts.

To neutralize HP-related stress impacts, microbial strains have different mechanisms. For instance, piezophiles such as *Photobacterium profundum* SS9 has a pressure-regulated ompH gene in a promotor that is responsible for elevated pressure-inducible transcription under high HP conditions [140]. Correspondingly, another microorganism, *Shewanella piezophila*, also has separate dynamic respiratory systems, acting at high and low pressure, respectively [141]. The presence of these respiratory chains in saline and osmolyte conditions leads to intracellular agglomeration of piezolytes along with ectoine agglomeration [127], N-trimethylamine oxide (TMAO) [128], and β-hydroxybutyrate [142]. In *Alcanivorax borkumensis*, under saline and osmolyte conditions, gene upregulation and intracellular ectoine accumulation were related to increased culture activity, diminished cell damage, and higher cell numbers [10,143].

### 5.3. Temperature

**Figure 3:**
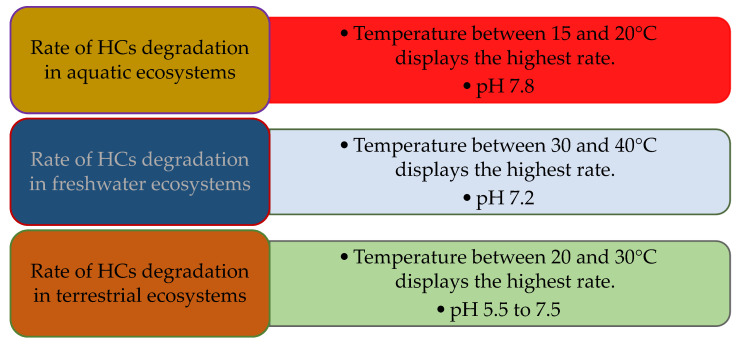
The optimal temperatures and pH for the highest HCs degradation in three ecosystems.

Among environmental factors, temperature plays a significant role in HCs biodegradation through influencing the physiology, diversity of the microbial flora, and the chemistry of the pollutants [144]. The ambient temperature of the ecosystem changes both the characteristics of the microbial activity and spilled oil. Although biodegradation of the HCs is possible over a wide range of temperatures (Figure 3), the biodegradation rate generally decreases with the reducing temperature (15–20 °C is the best range for marine environments) [145]. We witness an increase in the oil viscosity at lower temperatures, while the volatility of the toxic low molecular weight HCs is decreased, delaying the onset of biodegradation. The low temperature of marine environments can influence the solubility and saturation of fatty acids. Additionally, at lower temperatures, the rate of degradation decreases owing to enzymatic activity rates [32,90].

### 5.4. Microbial Community

Another factor affecting oil spill degradation is related to the composition and diversity of the microbial community. As indicated above, hydrocarbon degradation seems a versatile property of microbial communities. Consequently, specific and prevalent HCs-degrading microorganisms have been not identified by molecular techniques like 16S rRNA gene sequencing [110]. Even so, hydrocarbon availability selects for certain microbial groups: Upon chronic pollution, catabolic diversification occurs leading to the prevalence of both aerobic and anaerobic hydrocarbon degradation pathways in marine and ocean sediments [146]. Consequently, locations with chronic pollution have microbial strains with various catabolic abilities that give them a higher potential to degrade oil spills quickly [147].

The use of microorganisms in oil spill clean-up approaches in marine environments requests a proper knowledge of cooperation, competition, and succession among microbial communities. Microbial interactions in crude oil spillage highly depend on the microbial composition, which in turn is affected by environmental factors. For instance, metagenomic studies of polluted sediments from the Mediterranean to the Red Sea showed that metabolic pathways are influenced by the yearly average sediment temperature [148]. According to metagenomic data and isotope probing, the cooperation of different microbial community pathways is essential for PAH degradation abilities [149].

During the past decade, many studies have mechanistic modeling approaches, such as Community-Level Multispecies Flux Balance Analysis (FBA) have been introduced to predict growth yields and cell numbers of individual species, for a wide range of microbial interactions [150]. These models help to design new biodegradation strategies, to understand such microbial interactions during oil-degradation, and show fluxes through species. These modelling approaches still have some challenges. For instance, cell numbers of individual species are not provided, while this is of major interest to microbial ecologists to understand and predict community structure. Another major challenge is that multispecies FBAs predict up to a maximum of three interacting species while thousands of species can interact in just one milliliter of contaminated seawater [151]. Sequencing of 16S rRNA genes give insights in how the species may metabolically interact [152] and to construct microbial networks. We may also improve our knowledge on the organization of microbial interaction networks through systematic literature mining [153]. For example, a network based on published bacteria co-occurrences revealed that this network clustered into species groups that showed relations between resource competition, metabolic yield, and growth rate [151].

### 5.5. Product Toxicity of HCs Spills

Toxic metabolites originating from HC may seem the least significant factor. However, it is a vital factor that should be considered in the biodegradation process. For example, the PAHs concentration is one of the main marine pollutants and induces a selective pressure in microbes to react against PAHs by inducing changes in the structure of cell membrane, sporulation, and mycelia pigmentation [154,155]. Toxic compounds like oxy-PAHs (PAH-ketones and quinones) produced by the metabolism of microbes can contaminate the environment and affect many plants and animal aquatic species. To this date, the treatment or removal of oxy-PAHs from the environment is being performed through giant bioreactors; an ex situ controlled system for efficient oxy-PAH degradation [90]. Biostimulation with additional nutrients, bioaugmentation with useful microbes, and addition of non-ionic surfactants boost PAH bioremediation in bioreactors [156]. However, in situ degradation by adapted microbial communities might be a very suitable alternative.

## 6. Anaerobic and Aerobic Degradation of HCs and Involving Enzymes

A large number of microorganisms, including fungi, bacteria, green algae, and cyanobacteria have the potential of degrading various components of HCs under environmental conditions (e.g., pH, anaerobic and aerobic, and salinities). HCs degradation gradually happens by sequential metabolism of its compounds. Plasmid and/or chromosomal DNA is the main location of genes associated with HCs biodegradation. HCs biodegradation such as aromatic compounds and aliphatic can occur under both aerobic and anaerobic circumstances [157]. Using various terminal electron acceptors, the anaerobic degradation is catalyzed by anaerobic microorganisms like sulfate-reducing bacteria. Oxygen atoms are introduced into HCs by oxygenase enzymes, under aerobic conditions (generally, dioxygenases introduce two oxygen atoms whereas mono-oxygenases introduce only one oxygen). Owing to the metabolic merit of having the availability of oxygen as an electron acceptor, usually aerobic HCs catabolism is faster than anaerobic. Nonetheless, in some cases (e.g., limitations in oxygen availability) anaerobic degradation is also vital to the bioremediation process in environments like sludge digesters, and mangroves [157]. Aromatic compounds are hydrolyzed into benzoyl-CoA in anaerobic metabolism, which is the target of benzoyl-CoA reductase. Considering the different environmental conditions, various terminal electron acceptors may be applied, like nitrate, Fe (III), and sulfate [157].

Acetyl-CoA and electrons in the electron transport chain (This chain is constantly repeated and supplies further HCs degradation, and is normally completely oxidized to dioxide carbon) are the final products of saturated aliphatic HCs oxidation, which is catabolized in the citric acid cycle [158]. Aerobic conditions allow for toluene, xylene, benzene, and naphthalene degradation. The formation of catechol or a structurally related compound that is introduced in the citric acid cycle is a result of these compound degradations.

Among many various species of microbes, alkane hydroxylases are alkane-degrading enzymes in biodegradation [159]. Van Beilen and Funhoff argued that microbes use various ranges of alkanes, and that the final product of this degradation is alcohol. [160]. They suggested three categories of alkane-degrading enzyme systems including C17+, C1–C4, and C5–C16. Generally, the C17+ category includes longer alkanes oxidized by unknown enzyme systems. In C1–C4 as the second category, where methane to butane are oxidized via methane-monooxygenase-like enzymes. Finally, cytochrome P450 or integral membrane nonheme iron enzymes provide an opportunity for methane to butane oxidation in C1–C4 [160]. Subsequently, this research team reported unique methane monooxygenase (pMMO), cofactors, substrate, bacterial P450 oxygenase system, dioxygenase (CYP153, class I), AlkB-related alkane hydroxylases, and eukaryotic P450 (CYP52, class II) [160]. One of the most studied pathways encoded by the OCT plasmid belongs to Pseudomonas putida Gpo1 and converts an alkane into alcohol. For gram-negative bacteria, one visualizing model has been proposed by van Hamme indicating the ALK gene function and locations [161]. Regarding anaerobic aromatic HCs degradation and engaging enzymes, the catechol dioxygenase class is the best example, which is responsible for aromatic ring cleavage in bacteria with aromatic compounds degradation ability. This class catalyzes directly the molecular oxygen atoms to alcohol (1,2-dihydroxybenzene), with subsequent cleavage of the aromatic ring [159].

## 7. Conclusions

Oil spills bring many environmental and economic problems for humans and animals whose lives are tied to the marine environment. Among available degradation methods for deep-sea water, biodegradation techniques including biostimulation, biosurfactant addition, and bioaugmentation are the most affordable and efficient approaches. These bioremediation techniques demand a comprehensive understanding of factors engaging the growth, functions, and metabolism of indigenous microbial communities, at sites polluted with the HCs. The combination of omics including proteomics, metabolomics genomics, and transcriptomics, has presented an insight into microbial communities and their mechanisms in bioremediation. Based on a review of drivers of the biodegradation of HC compounds, we show how the effectiveness of these methodologies can be improved. These include the restoration of nutrient imbalances, creating electron flows by the ‘oil-spill snorkel’, consideration of microbial strains involved in the biodegradation processes as well as the interactions among microbial strains and the extent to which these interactions can be utilized to improve biodegradation of oil spills. In the near future, new explorations in microbial physiology and ecology characteristics related to HCs contamination in deep-sea water will likely provide more sustainable and innovative biology-based methods for the degradation of crude oils affected surface of deep-sea waters.

## Figures and Tables

**Table 1:** The list of investigated HCs-degrading microbial strains with their ecological and phylogeny features.

A/AN	Genome	Microorganism	Phylogeny	Target Substrate	Habitat	References
A	Y	*Alcanivorax borkumensis*	*γ-proteobacteria, Alcanivoracaceae*	*n*-alkanes	Seawater, sediment, beach sand, coastal salt marsh	[8,9]
A	Y	*Alcanivorax dieselolei*	*γ-proteobacteria, Alcanivoracaceae*	*n*-alkanes	Seawater, sediment	[10]
A	Y	*Marinobacter hydrocarbonoclasticus*	*γ-proteobacteria, Alteromonadaceae*	n-alkanes, PAHs	Seawater, sediment	[11]
A	Y	*Cycloclasticus pugetii*	*γ-proteobacteria, Piscirickettsiaceae*	PAHs	Sediment	[12,13]
A	Y	*Oleispira Antarctica*	*γ-proteobacteria, Oceanospirillaceae*	*n*-alkanes	Seawater	[14]
A	N	*Oleibacter marinus*	*γ-proteobacteria, Oceanospirillaceae*	*n*-alkanes	Seawater	[15]
A	N	*Oleiphilus messinensis*	*γ-proteobacteria, Oleiphilaceae*	*n*-alkanes	Seawater, sediment	[11]
A/AN	Y	*Pseudomonas pachastrellae*	*γ-proteobacteria, Pseudomonadaceae*	*n*-alkanes, PAHs	Sediment, beach sand	[16,17]
A/AN	Y	*Pseudomonas stutzeri*	*γ-proteobacteria, Pseudomonadaceae*	*n*-alkanes, PAHs, BTEX	Seawater, marine sediments, beach sand	[18]
A	N	*Halomonas halodurans; Halomonas organivorans*	*γ-proteobacteria, Halomonadaceae*	*n*-alkanes	Seawater, sediment	[19,20]
A	Y	*Thalassolituus oleivorans*	*γ-proteobacteria, Oceanospirillaceae*	*n*-alkanes	Surface seawaters, sediments, coastal and estuarine areas	[21]
A	Y	*Alteromonas naphthalenivorans*	*γ-proteobacteria, Alteromonadaceae*	PAHs	Seawater, tidal flat sediment	[22]
A	Y	*Acinetobacter venetianus*	*γ-proteobacteria, Moraxellaceae*	*n*-alkanes	Surface water, sediment.	[23]
A	Y	*Dietzia maris*	*Actinobacteria, Dietziaceae*	*n*-alkanes, PAHs	Seawater, deep sea hydrothermal field	[24]
A	N	*Rhodobacter* sp. *SS12.29; Rhodococcus* sp. *ice-oil-488 s*	*γ -proteobacteria, Rhodobacteraceae*	PAHs	Seawater	[25]
A	N	*Sphingopixis* sp.	*γ -proteobacteria, Sphingomonadaceae*	PAHs	Seawater	[25]
AN	Y	*Desulfatibacillum alkenivorans*	*γ -proteobacteria, Desulfobacteraceae*	*n*-alkanes	Sediment	[26]
AN	N	*Desulfosarcina-Desulfococcus cluster strains*	*γ -proteobacteria, Desulfobacteraceae*	Short chain *n*-alkanes	Sediments of marine HC seeps	[2,27]
AN	N	*Desulfococcus oleovorans*	*γ -proteobacteria, Desulfobacteraceae*	*n*-alkanes, aromatic HCs	Sediment	[28]
A	Y	*Bacillus pumilus*	*Bacilli, Bacillaceae*	*n*-alkanes, PAHs	Sediment	[18,29]
A	N	*Bacillus stratosphericus*	*Bacilli, Bacillaceae*	PAHs, BTEX	Seawater	[6]
AN	Y	*Archaeoglobus fulgidus*	*Euryarchaeota, Archaeoglobaceae*	*n*-alkanes	Shallow marine hydrothermal system	[30]
AN	Y	*Thermococcus sibiricus*	*Euryarchaeota, Thermococcaceae*	*n*-alkanes	Oil reservoir	[31]
AN	Y	*Ferroglobus placidus*	*Euryarchaeota, Archaeoglobaceae*	Aromatic HCs	Shallow marine hydrothermal system	[32,33]
AN	N	*Dothideomycetes-related taxa*	*Fungi*	PAHs	Beach sediment, tarballs, salt marshes	[34,35]

Y—Yes genome available for at least one strain, N—Not available genome (Genome availability checked on NCBI database on 27 November 2016), A—Aerobic, AN—Anaerobic, PAH—Polycyclic Aromatic Hydrocarbons, BTEX—Benzene, Toluene, Ethyl Benzene and Xylene.

**Table 2:** Different sources of PAHs.

Source	Examples		
Mobile Sources	Vehicle Exhausts [55]	Aircraft Exhaust [56]	Oil Tankers [57]
Industrial Sources	Coke Production/Burning [58]	Cement Manufacturing [59]	Tyre Manufacturing [58]
Domestic Sources	Coal Cooking [56]	Wood Burning [55]	Cigarette/Tobacco Smoking [60]
Agricultural Sources	Agricultural Wastes [55]	Pesticides [61]	Fertilizers [61]
Natural Sources	Forest Fire [62]	Volcanic Eruptions [63]	Wild Fire [63]

**Table 3:** Microbial consortia in bioaugmentation.

Pollutant Type	Microorganisms	Reference
PAHs (fluorene, pyrene, phenanthrene)	*Rhodococcus* sp*., Acinetobacter* sp*., Pseudomonas* sp.	[78]
Gasoline	*Methylibium petroleiphilum* LMG22953	[79]
Crude oil	*Roseomonas* sp*., Bacillus marisflavi, Microbacterium oxydans*	[80]
Crude oil	*Alcanivorax borkumensis, Thalassolituus oleivorans*	[81]
Crude oil	*P. aeruginosa, Rhodococcus* sp*. CE461, Rhodococcus* sp*. CT451*	[82]
Petroleum HCs	*Rhizopus* sp*., Penicillium funiculosum, Aspergillus sydowii, Rhizobiales* sp*., Pseudomonas* sp.*, Brucella* sp.*, Bacillus* sp.*, Rhodococcus* sp., *Microbacterium* sp.	[83]
Petroleum HCs	*Pseudomonas oleovorans, Ochrobactrum* sp.*, Stenotrophomonas maltophila*	[84]
Mixture of PAHs (anthracene, naphthalene, phenanthrene, pyrene, dibenzo[a]anthracene	*Bacillus strains* B1F, B5A and B3G*, Chromobacterium* sp. 4015*, Enterobacter aglomerans* sp. B1A	[85]
PAHs (anthracene, phenanthrene, pyrene)	*Mycobacterium fortuitum, Bacillus cereus, Microbacterium* sp., *Gornodia, Polyisoprenivorans, Microbacteriaceae, Bacterium, Fusarium oxysporium*	[86]
Crude petroleum oil hydrocarbon	*B. subtilis* DM-04*, P. aeruginosa M and* NM	[87]

**Table 4:** Economic and prevalent biosurfactants in the bioremediation process.

Microorganisms	Biosurfactant	Economic Significance	References
*P. aeruginosa*	Rhamno lipids	Bioremediation	[102]
*Acinetobacter calcoaceticus*	Emulsan Glycolipopeptide	Enhanced oil recovery by microbes	[103]
*Rhodococcuserythropolis*	Trehalose lipids	Dissolution of HCs	[104]
*Ustilagomaydis*	Cellobiose lipids	Antifungal compounds	[105]
*Microbacterium*	Microbactan Glycolipopeptide	Emulsifier	[106]
*B. licheniformis*	Lichenysin	Enhanced oil recovery by microbes	[107]
*C. bombicola*	Sophoro lipids	Antimicrobial property	[108]
*B. subtilis*	Surfactin	Antimicrobial property	[109]

## Data Availability

All data, tables, figures and results in paper are our own and original.
